# Differential adaptive RNA editing signals between insects and plants revealed by a new measurement termed haplotype diversity

**DOI:** 10.1186/s13062-023-00404-7

**Published:** 2023-08-17

**Authors:** Yuange Duan, Ye Xu, Fan Song, Li Tian, Wanzhi Cai, Hu Li

**Affiliations:** Key Laboratory of Pest Monitoring and Green Management, Department of Entomology, College of Plant Protection, China Agricultural University, Beijing, 100193 China

**Keywords:** RNA editing, *Arabidopsis thaliana*, Insect, Nonsynonymous, Haplotype diversity, Restorative

## Abstract

**Background:**

C-to-U RNA editing in plants is believed to confer its evolutionary adaptiveness by reversing unfavorable DNA mutations. This “restorative hypothesis” has not yet been tested genome-wide. In contrast, A-to-I RNA editing in insects like *Drosophila* and honeybee is already known to benefit the host by increasing proteomic diversity in a spatial-temporal manner (namely “diversifying hypothesis”).

**Methods:**

We profiled the RNA editomes of multiple tissues of *Arabidopsis thaliana*, *Drosophila melanogaster*, and *Apis melifera*. We unprecedentedly defined the haplotype diversity (HD) of RNA molecules based on nonsynonymous editing events (recoding sites).

**Results:**

Signals of adaptation is confirmed in *Arabidopsis* by observing higher frequencies and levels at nonsynonymous editing sites over synonymous sites. Compared to A-to-I recoding sites in *Drosophila*, the C-to-U recoding sites in *Arabidopsis* show significantly lower HD, presumably due to the stronger linkage between C-to-U events.

**Conclusions:**

C-to-U RNA editing in *Arabidopsis* is adaptive but it is not designed for diversifying the proteome like A-to-I editing in *Drosophila*. Instead, C-to-U recoding sites resemble DNA mutations. Our observation supports the restorative hypothesis of plant C-to-U editing which claims that editing is used for fixing unfavorable genomic sequences.

**Supplementary Information:**

The online version contains supplementary material available at 10.1186/s13062-023-00404-7.

## Background

### RNA editing in tree of life

RNA editing is prevalent in all kingdoms of lives [1–3]. Traditionally, RNA editing includes the adenosine-to-inosine (A-to-I) RNA editing in animals mediated by ADARs (adenosine deaminase acting on RNA) [4, 5] and the cytidine-to-uridine (C-to-U) RNA editing in plants mediated by factors like PPR (pentatricopeptide repeat) proteins [6]. Although C-to-U editing also exists in animals, the numbers of such transitions are negligible [7]. Since I is read as G in the cell system, both A-to-I and C-to-U RNA editing could recode the CDS (coding sequence) and change protein sequence. The recoding events are termed nonsynonymous editing (Nonsyn) and the other silent editing events in CDS are termed synonymous editing (Syn).

### Two complementary hypotheses on the significance of nonsynonymous editing

RNA editing has various biological functions. Apart from the non-coding RNA editing sites that are dedicated to immune and anti-viral responses [8], the many RNA editing events in CDS, especially the nonsynonymous editing sites, have two major roles. Two complementary hypotheses explain the evolutionary significance of nonsynonymous editing sites at genome-wide level. (1) The “diversifying hypothesis” believes that RNA editing is adaptive as it flexibly increases the proteomic diversity in a spatial-temporal manner [9]. By RNA editing, organisms can selectively recode the protein sequence whenever needed, avoiding the pleotropic effect of DNA mutations [10]. (2) The “restorative hypothesis” claims that nonsynonymous RNA editing events are designed for reversing unfavorable DNA mutations [11]. For example, A-to-I(G) RNA editing can reverse recent G-to-A DNA mutations and restore the ancestral allele (G). Under this circumstance, given that the ancestral DNA allele (G) is optimal, the currently edited allele is still “no fitter than” the ancestral state so that the editing events are interpreted as non-adaptive [11]. However, undoubtedly, edited allele (G) in present species is fitter than the unedited allele (A) and therefore the editing mechanism itself should be adaptive.

### Nonsynonymous C-to-U RNA editing in plants

In plants, C-to-U RNA editing events take place in chloroplast and mitochondrial genes. These RNA editing events are mediated by various factors. These factors include the first discovered site-specific editing/splicing factors PPRs that recognize and bind to target RNA regions [12], the secondly discovered multiple organellar RNA editing factor (MORF) that exerts RNA editing in mitochondria of angiosperms [13], organelle RNA recognition motif-containing (ORRM) that forms complexes with other editing factors [14], protoporphyrinogen IX oxidase 1 (PPO1) and RanBP2-type zinc finger protein family member organelle zinc finger 1 (OZ1) that are responsible for several plastid editing sites [15, 16]. Many of these factors collaborate with each other to form complexes and collectively trigger the RNA editing events [14, 16–18]. However, at genome-wide level, the evolutionary significance of C-to-U RNA editing sites was not as well-understood as the editing machinery.

Lines of evidences show that the C-to-U RNA editing events (mainly in non-nuclear genome) conform to the restorative hypothesis [19]. This means that C-to-U editing should mimic C-to-T DNA mutations to reverse recent T-to-C mutations. To achieve the restorative effect by resembling DNA mutations, the C-to-U RNA editing events should:

(1) Bear high editing levels. Since T allele is optimal and C allele is non-optimal under the restorative hypothesis, the C-to-U editing at a particular site should achieve as high level as possible. For DNA mutations (homozygote), the alternative allele level is 100%. Similarly, natural selection would force the RNA editing level to increase. Note that this rule is only for nonsynonymous sites because we presume that synonymous sites are neutral. Moreover, we should also explain that no strict criteria could define what is the value of being high. The restorative hypothesis is a new theory which was first proposed in 2019 [11] and was continuously undergoing extensive debate [20]. High and low should be defined as a relative value [21].

(2) RNA editing should have globally low tissue-specificity. DNA mutations have no tissue-specificity at all, neither does C-to-U RNA editing under the restorative hypothesis. If only some tissues need to be restored by C-to-U editing, then this scenario will instead support the diversifying hypothesis that stresses the spatial-temporal flexibility of RNA editing. Note that a globally low tissue-specificity required by restorative hypothesis still allows particular editing sites to be tissue-specific because all evolutionary theories are described at genome-wide level and do not focus on special cases. For example, 41 organelle editing sites were reported to have tissue-specificity between roots and leaves of tobacco (*Nicotiana tabacum*) [22]. However, this only accounts for a small fraction of the totally identified ~ 500 unique sites. Moreover, in Kiwifruit (*Actinidia chinensis*), a few editing sites solely occur in leaves [23]. Again, these special cases of tissue-specific editing do not deny the overall pattern that the majority of editing sites in plants would keep a constantly high level across different tissues [24]. Accordingly, as we will show in our results, the tissue-specificity of plant RNA editing is much lower than the tissue- or stage-specificity of RNA editing in insects.

### Hidden evidences that support the restorative effect of C-to-U editing

The above-mentioned two implications of the restorative hypothesis of C-to-U RNA editing have been sporadically observed by different studies [2, 25–29]. However, those are merely limited observations (or case studies) demonstrating the restorative effect of C-to-U editing. Observations need to be refined to create a general rule. To date, no one has ever drawn a formal conclusion about the restorative role of C-to-U editing at genome-wide level. Here, we will find evidences to support the restorative hypothesis.

Importantly, since the restorative hypothesis and diversifying hypothesis are mutually exclusive, proving that C-to-U editing does not belong to the latter (diversifying) will increase the confidence that it belongs to the former (restorative). Interestingly, it is known that A-to-I RNA editing in insects like *Drosophila* plays a diversifying role [10, 30]. If we prove that C-to-U editing in plants has less power to increase diversity compared to A-to-I editing in insects, then this will support the notion that plant C-to-U editing plays a restorative role.

### Haplotype diversity (HD) describes the proteomic diversity achieved by nonsynonymous editing

**Fig. 1:**
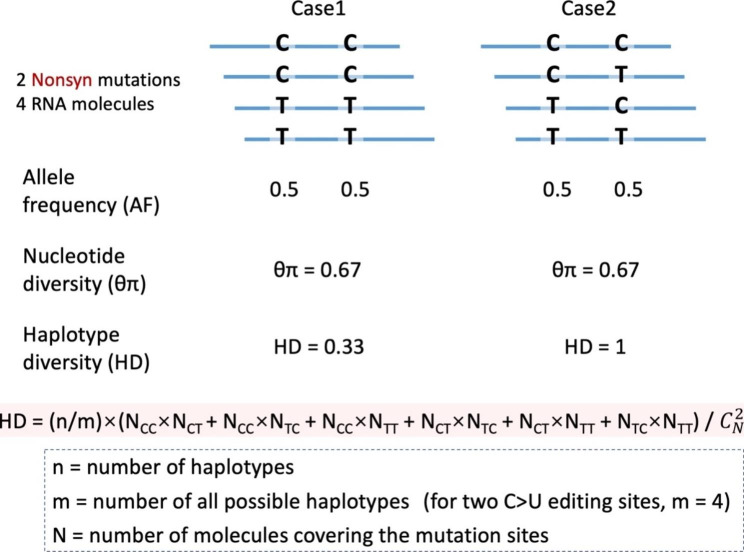
Definition of haplotype diversity (HD). Each haplotype is treated as a different element to calculate the pairwise difference among the haplotypes. For two nonsynonymous (Nonsyn) mutations or RNA editing sites, the HD would represent the proteomic diversity because each haplotype produces a different protein isoform

To measure the proteomic diversity created by nonsynonymous editing sites, we defined haplotype diversity (HD). The traditional nucleotide diversity parameter θπ (pairwise nucleotide difference) does not consider the linkage between mutations. For example, case1 (4 molecules, genotype: CC, CC, TT, TT) and case2 (4 molecules, genotype: CC, CT, TC, TT) have identical θπ = 0.67 (Fig. 1). However, case1 has only 2 haplotypes while case2 has 4 haplotypes. Case2 obviously has higher diversity than case1.

Here, we define haplotype diversity HD as the pairwise difference of a region. HD algorithm regards every haplotype as a different element (see Materials and Methods for details). If we only consider nonsynonymous mutations, then each haplotype (with different combinations of mutations) will produce a different protein isoform. Thus, haplotype diversity HD equals the proteomic diversity. For example, for the two nonsynonymous C-to-U(T) editing sites, case1 has HD = 0.33 while case2 has HD = 1 (Fig. 1). HD successfully improves θπ by considering the linkage information between mutations and faithfully reflects the proteomic diversity caused by nonsynonymous mutations.

**Fig. 2:**
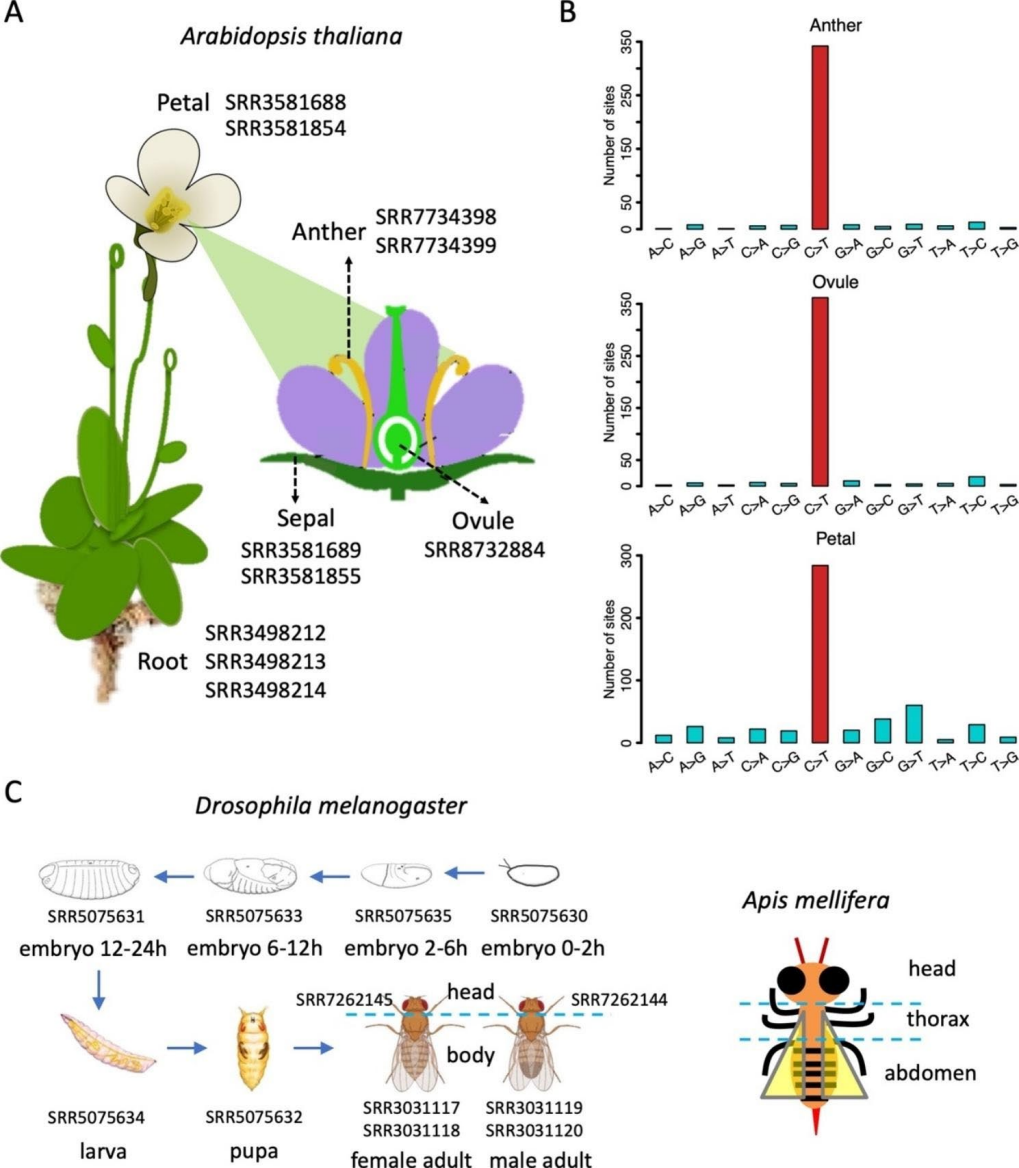
Tissues and accession numbers of *A. thaliana* samples and their variant profile. (**A**) RNA-Seq data from five *A. thaliana* tissues and the accession ID: anther (SRR7734398 & SRR7734399), mature ovule (SRR8732884), petal of mature flower (SRR3581688 & SRR3581854), sepal of mature flower (SRR3581689 & SRR3581855), and root (SRR3498212, SRR3498213, SRR3498214). (**B**) The transcriptomes of anther, ovule, and petal exhibit significant enrichment of C-to-T mutations, representing C-to-U RNA editing sites. C > T means C-to-T mutations, the same goes for other types of variations. (**C**) RNA-Seq data of 10 developmental stages/tissues of fruitfly *Drosophila melanogaster* and 3 tissues of honeybee *Apis mellifera*

In this study, we retrieved RNA-Seq data from multiple tissues of *Arabidopsis thaliana* (Fig. 2A). By comparing the haplotype diversity HD of C-to-U editing in *A. thaliana* to that of A-to-I editing in fly brains, we found that plant C-to-U editing obviously has lower HD than *Drosophila* A-to-I editing. Our work is the first study that systematically investigates RNA editomes from the angle of haplotype diversity. Our results strongly support previous notion that A-to-I editing in flies is designed for diversifying purpose and also serve as evidence proving that C-to-U editing in plants is used for restoration rather than diversifying the proteome.

## Materials and methods

### Data collection

We downloaded the RNA-Seq data of different tissues of *Arabidopsis thaliana* from NCBI with the following accession IDs: anther (SRR7734398 & SRR7734399), mature ovule (SRR8732884), petal of mature flower (SRR3581688 & SRR3581854), sepal of mature flower (SRR3581689 & SRR3581855), and root (SRR3498212, SRR3498213, SRR3498214). The reference genome of *A. thaliana* was downloaded from Ensembl (genome version TAIR10). The A-to-I RNA editing sites in brains of *Drosophila melanogaster* were retrieved from our previous work [10]. The mRNA-Seq data of developmental stages and tissues of *D. melanogaster* were downloaded from NCBI with the following accession numbers: embryo 0-2 h (SRR5075630), embryo 2-6 h (SRR5075635), embryo 6-12 h (SRR5075633), embryo 12-24 h (SRR5075631), larva (SRR5075634), pupa (SRR5075632), female adult body (SRR3031117 & SRR3031118), male adult body (SRR3031119 & SRR3031120), female adult head (SRR7262145) and male adult head (SRR7262144). The honeybee (*Apis mellifera*) data and the matched editing sites were retrieved from our previous work [30].

### Mapping, variant calling, and C-to-U RNA editing sites

Since C-to-U RNA editing in plants mainly occur in chloroplast and mitochondrial genes, RNA-Seq reads were directly mapped to the CDS of chloroplast and mitochondrial genes of *A. thaliana*. Bowtie2 [31] under default parameters was used to align the reads. Variants were called using samtools mpileup v1.11 [32]. Nucleotides with base quality lower than 30 were discarded in the variant calling procedure (-Q 30), which means that the remaining bases would have error rates lower than 0.001. For example, if a site has 1000 reads covered and only one read supports the mutation (while 999 reads support the reference allele), this mutation is still reliable as the error rate of the bases is already less than 0.001. The golden standard for accurate editing detection is to achieve a high fraction of the desired type of mutation (for example, C-to-T). This standard, as long as the C-to-T fraction is high, does not necessarily require particular cutoffs on sequencing depth or alternative reads count. For example, in the field of animal A-to-I(G) RNA editing, a widely used pipeline was developed to identify hyper-editing sites solely based on the A-to-G fraction regardless of other cutoffs [33, 34]. Similarly, in this study, we used the samples (tissues) which performed well in the C-to-T enrichment to anchor the candidate C-to-U editing sites. For the remaining samples, we directly investigated the editing status on these positions.

Among the five samples (tissues) used in this study, anther, ovule, and petal have remarkably high C-to-T fractions, suggesting reliable C-to-U RNA editing sites. These three samples were used to anchor editing sites. Totally 507 unique C-to-U sites were obtained. Then, for all samples, we calculated the alternative reads count (T), reference reads count (C), sequencing depth (C + T), and editing levels = T/(T + C).

The linkage disequilibrium (LD) between C-to-U RNA editing sites was done by sam2tsv plus R script following our previous pipeline [35]. The LD information including the haplotype frequency is used for calculating the haplotype diversity (see below). The A-to-I RNA editing sites in brains of *D. melanogaster* and the linkage information were retrieved from our previous works [10, 35].

### A-to-I RNA editing sites in flies and bees

A-to-I RNA editing sites in *Drosophila melanogaster* (10 samples as described) were based on known sites recorded by previous studies and datasets [10, 36–40]. Totally 7,422 unique candidate editing sites were obtained. Samtools mpileup v1.11 (-Q 30) [32] was used to extract the editing status (reference allele count and alternative allele count) on each candidate editing sites. We maintained the sites with editing events detected in at least one sample, resulting in 2,345 unique final editing sites in these samples. These editing sites included 1,008 nonsynonymous and 284 synonymous sites.

The honeybee (*Apis mellifera*) data and the editing sites information were obtained from our previous study [30]. There were totally 407 editing sites including 111 nonsynonymous and 9 synonymous sites.

### Annotation of nonsynonymous and synonymous editing sites

For each C-to-U editing site in a codon, we examined whether this C-to-U(T) mutation will alter the amino acid. Amino acid-changing mutations are nonsynonymous and the remaining C-to-U editing sites in CDS are synonymous. Among the 507 unique C-to-U editing sites, 402 sites were nonsynonymous and 105 sites were synonymous.

### Expected nonsyn/syn ratio and signals of adaptation

If we manually change all Cs to Us(Ts) in the CDS of chloroplast and mitochondrial genes, we will obtain 19,149 nonsynonymous and 9,459 synonymous mutations. The Nonsyn/Syn ratio (19,149/9,459 = 2.02) is regarded as the expected Nonsyn/Syn ratio. The difference between observed Nonsyn/Syn ratio of C-to-U editing and the expected Nonsyn/Syn ratio is calculated by Fisher’s exact test. O/E ratio (observed/expected) > 1 suggests positive selection on C-to-U RNA editing and thus shows signal of adaptation.

### Haplotype diversity (HD)

For each pair of nonsynonymous C-to-U(T) RNA editing sites, we calculated the haplotype diversity HD (Fig. 1).

HD = (n/m) × (N_CC_×N_CT_ + N_CC_×N_TC_ + N_CC_×N_TT_ + N_CT_×N_TC_ + N_CT_×N_TT_ + N_TC_×N_TT_) / $${C}_{N}^{2}$$.

Where n is the observed number of haplotypes, m is the total possible haplotypes. For two C-to-U(T) editing sites, there will be four possible haplotypes CC, CT, TC, and TT, then m = 4.

N_ij_ (i, j = C or T) is the number of reads supporting haplotype N_ij_.

${C}_{N}^{2}$ = N×(N-1)/2 is the number of combinations by choosing 2 reads from a total pool of N reads that cover the two C-to-U editing sites. N = N_CC_ + N_CT_ + N_TC_ + N_TT_.

We only consider nonsynonymous editing sites so that the haplotype diversity will reflect the proteomic diversity. In this study, we required a pair of sites to have N ≥ 5 to minimize the detection bias caused by low sequencing coverage.

If we calculate the traditional nucleotide diversity θπ on two editing sites, it will be: θπ = (N_CC_×N_CT_ + N_CC_×N_TC_ + 2×N_CC_×N_TT_ + 2×N_CT_×N_TC_ + N_CT_×N_TT_ + N_TC_×N_TT_) / (2×${C}_{N}^{2}$). This θπ parameter does not take linkage information into account and will underestimate the real haplotype diversity (proteomic diversity) if the proportion of 4 haplotypes are relatively even (Fig. 1).

### Definition of differential editing sites (DES)

Differential editing sites (DES) were defined between any two tissues of *A. thaliana*. Let “at”,“ov”,“pt”,“sp”,“rt” be anther, ovule, petal, sepal, and root. The R script for identifying DES is as follows. Take nonsynonymous editing sites for instance:

df_des_nonsyn <- as.data.frame(matrix(ncol = 5,nrow = 5)).

colnames(df_des_nonsyn) <- names(list_candi).

rownames(df_des_nonsyn) <- names(list_candi).

for(j in c(“at”,“ov”,“pt”,“sp”,“rt”)){.

for(k in c(“at”,“ov”,“pt”,“sp”,“rt”)){.

df1 <- list_candi[[j]];df2 <- list_candi[[k]].

df3 <- cbind(df1[df1$anno=="Nonsyn”,c(“C”,“A”)],df2[df2$anno=="Nonsyn”,c(“C”,“A”)])

df3$p <- apply(df3,1,function(x){.

x[1] <- x[1]-x[2];x[3] <- x[3]-x[4].

fisher.test(matrix(x,ncol = 2))$p.value.

})df3$fdr <- p.adjust(df3$p,method="fdr”).

df_des_nonsyn[j,k] <- sum(df3$fdr < 0.05).

}

}

The same goes for the code for synonymous editing sites.

In brief, for each editing site, the reads count numbers (reference allele count and alternative allele count) of two samples were extracted. Fisher’s exact tests were used to calculate the *P* value of the difference in editing level = alt/(ref + alt). Then the *P* values were adjusted for multiple testing correction to get a false discovery rate (FDR) [41]. Editing sites with FDR < 0.05 were regarded as DES. The same goes for DES among *D. melanogaster* and *A. mellifera* samples.

## Results

### C-to-U RNA editome of Arabidopsis thaliana shows signal of adaptation

We profiled the RNA editomes of different tissues of *A. thaliana* (Fig. 2A). The golden standard for editing identification is to obtain an enrichment of the target mutation type. Anther, ovule, and petal have impressively high C-to-T fractions among all 12 types of mutations (Fig. 2B). Therefore, these three samples were used to anchor the C-to-U editing sites (Materials and Methods). Then, as we will describe later, we also collected the RNA-Seq data of 10 developmental stages/tissues of fruitfly *Drosophila melanogaster* and 3 tissues of honeybee *Apis mellifera* (Fig. 2C and Materials and Methods) for further comparison between plants and insects.

**Fig. 3:**
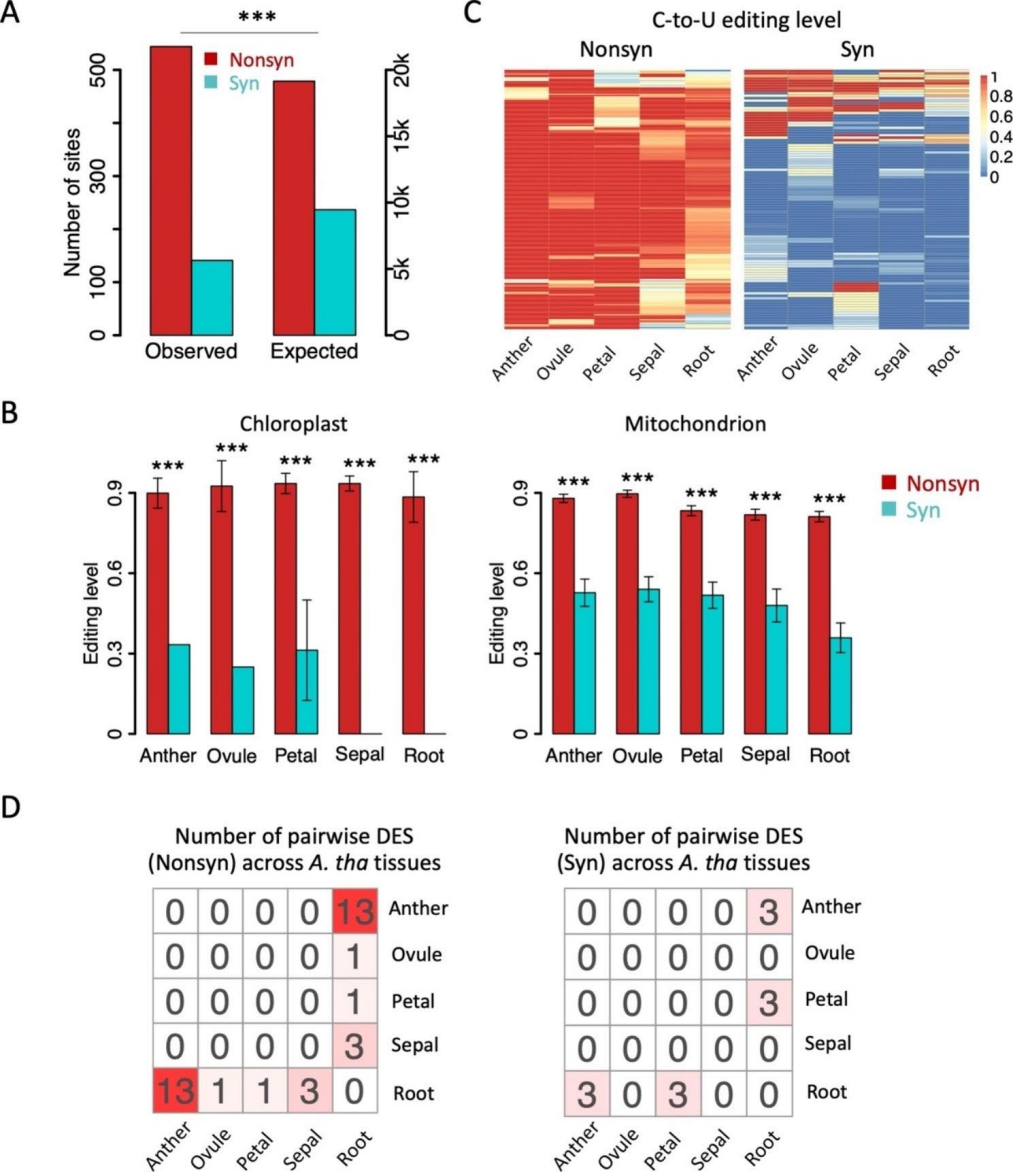
Signals of adaptation of the *A. thaliana* editomes. (**A**) The numbers of observed nonsynonymous and synonymous editing sites were compared to the random expectation under neutral evolution. *P* value was calculated by Fisher’s exact test. ***, *P* < 0.001. (**B**) Comparison of editing levels of nonsynonymous and synonymous editing sites in various tissues of *A. thaliana*. Error bars represent standard error of mean. *P* values were calculated with Wilcoxon rank sum tests. ***, *P* < 0.001. Chloroplast and mitochondrial editing sites were shown respectively. (**C**) Heatmaps displaying the conservation of editing levels of individual editing sites. Nonsynonymous editing sites obviously have less tissue-specificity than synonymous editing sites. (**D**) Pairwise differential editing sites (DES) between *A. thaliana* tissues. Nonsynonymous and synonymous sites were shown separately

Totally 507 unique C-to-U editing sites were obtained in CDS of *A. thaliana* (chloroplast and mitochondrial genes), including 402 nonsynonymous and 105 synonymous sites. The Nonsyn/Syn ratio is 402/105 = 3.8, which is significantly higher than random expectation (19,149/9,459 = 2.02) under neutral evolution (Fig. 3A and also see Materials and Methods). Notably, among the total 507 editing sites, 457 sites were in mitochondrial genes (Nonsyn/Syn = 358/99) while only 50 sites were in chloroplast genes (Nonsyn/Syn = 44/6). Moreover, the editing levels are also higher at nonsynonymous sites compared to synonymous sites for both chloroplast and mitochondrial editing sites (Fig. 3B). These patterns suggest that the nonsynonymous C-to-U editing sites in *A. thaliana* are generally adaptive and are positively selected. Next, we need to consider which adaptive hypothesis does plant RNA editing follow.

### Confirm the restorative hypothesis of plant RNA editing from different aspects

Although signals of adaptation were observed in the C-to-U editome of *A. thaliana*, the nature of the adaptiveness is still unknown. Both diversifying hypothesis [9, 10] and restorative hypothesis [11] require nonsynonymous editing to have higher occurrence and editing levels than synonymous editing. Therefore, the signals of adaptation do not help distinguish between the two hypotheses. The restorative hypothesis proposes that nonsynonymous RNA editing should mimic DNA mutations while the diversifying hypothesis emphasizes the increase of proteomic diversity by RNA editing. Intuitively, the restorative hypothesis would imply that nonsynonymous editing levels should be sufficiently high and lack tissue specificity. We have just observed that C-to-U editing levels in *A. thaliana* are high (especially for nonsynonymous sites) (Fig. 3B). Here, we further confirmed that nonsynonymous C-to-U editing levels lack tissue-specificity (Fig. 3C), suggesting that C-to-U RNA editing resembles DNA mutation (at least for nonsynonymous editing sites) in many aspects. To quantitatively show the extent of tissue-specificity of RNA editing, we carried out a measurement to define the pairwise differential editing sites (DES) between two samples (Materials and Methods). Among the 402 nonsynonymous editing sites, we only found 13 DES between anther and root, and 5 DES across other tissues (Fig. 3D). The average fraction of pairwise DES is 18/${\text{C}}_{5}^{2}$/402 = 0.45%. Among the 105 synonymous editing sites, only 6 DES were found across different tissues (Fig. 3D) and the average fraction of pairwise DES is 6/${\text{C}}_{5}^{2}$/105 = 0.57%. The purpose of calculating this fraction is to make a comparison between the extent of tissue (or sample)-specificity of RNA editing events in plants *versus* animals (insects).

The evidences demonstrated in this section would support the predictions made by restorative hypothesis. Note that although the restorative purpose of specific C-to-U editing sites has already been proposed by early studies [2, 25–29], genome-wide evidence was not provided by them.

### Insect nonsynonymous editing shows great tissue- or stage-specificity

**Fig. 4:**
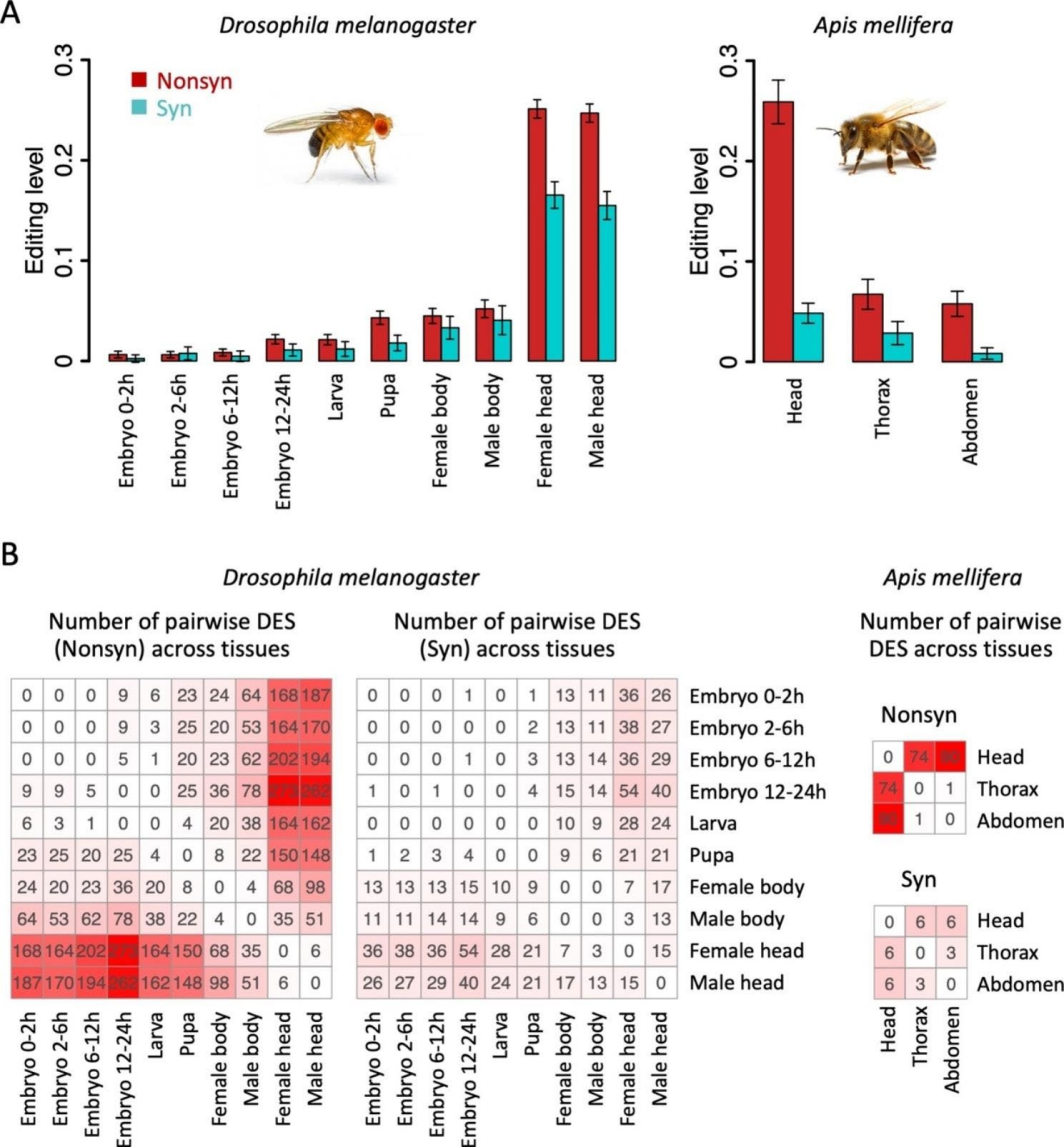
Tissue- or stage-specificity of A-to-I RNA editing in insects. (**A**) Editing levels of nonsynonymous and synonymous editing sites in various samples of *D. melanogaster* and *A. mellifera*. Error bars represent standard error of mean. (**B**) Pairwise differential editing sites (DES) among *D. melanogaster* and *A. mellifera* samples. Nonsynonymous and synonymous sites were shown separately

Next, we performed similar analyses to investigate the tissue- or stage-specificity of RNA editing sites in animals. We collected the transcriptome of 10 developmental stages/tissues of fruitfly *D. melanogaster* and 3 tissues of honeybee *A. mellifera* (Fig. 2C) and obtained the A-to-I RNA editing profile in each sample (Materials and Methods). Totally 1,008 nonsynonymous and 284 synonymous editing sites were found in fruitfly and 111 nonsynonymous and 9 synonymous editing sites were obtained in honeybee (Materials and Methods). Apart from the known fact that in insects, the nonsynonymous editing bore higher editing levels than synonymous editing sites (signals of adaptation), we additionally observed that both fruitfly and honeybee showed strong tissue- or stage-specificity on editing sites (Fig. 4A). The editing levels were highest in adult heads and almost ignorable in other tissues/stages.

We calculated the pairwise DES among the 10 fruitfly samples and 3 honeybee samples. Remarkably, in both insect species, head samples had many DES compared with other samples (Fig. 4B). We also calculated the average fraction of pairwise DES. This fraction was 3084/${\text{C}}_{10}^{2}$/1008 = 6.8% for *Drosophila* nonsynonymous editing and 585/${\text{C}}_{10}^{2}$/284 = 4.6% for *Drosophila* synonymous editing, and 165/${\text{C}}_{3}^{2}$/111 = 50.0% for honeybee nonsynonymous editing and 15/${\text{C}}_{3}^{2}$/9 = 55.6% for honeybee synonymous editing. These fractions were obviously higher than the ~ 0.5% obtained in 5 tissues of *A. thaliana*, suggesting that the tissue-specificity of plant RNA editing was extremely low, at least for *A. thaliana*. Note that this fraction in *Drosophila* (6.8% and 4.6%) seems much lower than the faction in honeybee, but we should notice that *Drosophila* has 10 samples so the pairwise DES number is diluted by the combination number ${\text{C}}_{10}^{2}$ = 45 (while this combination number is only 3 for honeybee). Anyway, the insect RNA editing has much stronger tissue-specificity than plant RNA editing.

### C-to-U editing in A. thaliana shows low haplotype diversity

Philosophically, if group A and group B are mutually exclusive, then “X does not belong to group A” would increase the probability that “X belongs to group B”. Given that the diversifying hypothesis and restorative hypothesis are complementary, we intend to prove that C-to-U editing in *A. thaliana* has less power to increase proteomic diversity compared to the A-to-I editing in *Drosophila* (which is well-known for its diversifying role). By showing this comparison, it will be more confident to claim that C-to-U editing in plants is used for restorative purpose rather than diversifying purpose.

**Fig. 5:**
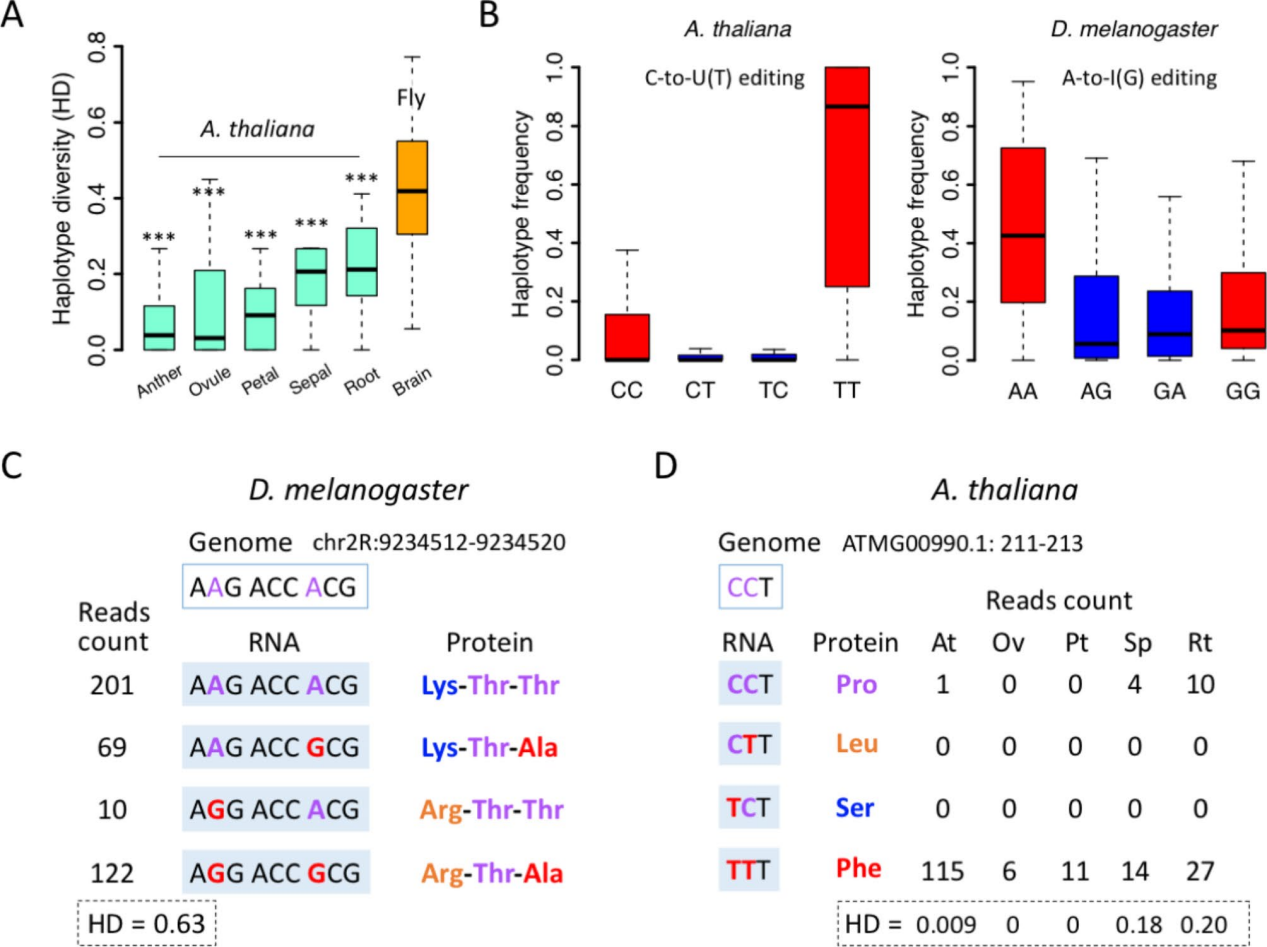
C-to-U(T) RNA editing in *A. thaliana* is less capable of diversifying the proteome compared to A-to-I(G) RNA editing in *D. melanogaster*. (**A**) Haplotype diversity (HD) of nonsynonymous editing sites. Five tissues of *A. thaliana* were compared to fly brains with Wilcoxon rank sum tests. ***, *P* < 0.001. (**B**) Haplotype frequency of the four possible combinations of two nonsynonymous editing sites. In *A. thaliana*, the lack of CT and TC haplotype will largely reduce HD. (**C**) A case of two nearby A-to-I(G) nonsynonymous editing sites in *D. melanogaster*. The reads count for each haplotype and the HD were shown. (**D**) A case of two adjacent C-to-U(T) nonsynonymous editing sites in *A. thaliana*. The reads count for each haplotype and the HD were shown. At, anther; Ov, ovule; Pt, petal; Sp, sepal; Rt, root

We calculated the haplotype diversity (HD) parameter for each pair of nonsynonymous editing sites (Fig. 1 and Materials and Methods). When measuring the diversity of protein isoforms, HD (on nonsynonymous sites) obviously performs better than the traditional nucleotide diversity θπ because θπ does not take into account the linkage information (Fig. 1 and Materials and Methods). Interestingly, we observed significantly lower HD in all tissues of *A. thaliana* compared to *Drosophila* brains (Fig. 5A). This suggests that the nonsynonymous C-to-U RNA editing in *A. thaliana* is indeed not designed for the diversifying purpose. Notably, based on our criteria aiming at obtaining high-confidence HD values, we required the pairs of nonsynonymous editing sites to have sufficient reads coverage (Materials and Methods). This resulted in 87, 56, 13, 12, and 42 pairs of nonsynonymous sites in the five *A. thaliana* tissues, respectively (Fig. 5A). All these pairs were located in mitochondrial genes so that the cases in chloroplast genes could not be tested under the current data size. Therefore, we should emphasize that the conclusion of our paper on HD mainly relies on mitochondrial genes. Moreover, this might also reflect a fact that the nonsynonymous editing sites in chloroplast genes are less clustered so that it is unlikely to find two sites covered by the same sequencing reads then the haplotype analysis could not be performed.

We wonder why RNA editing shows lower haplotype diversity in *A. thaliana* than in *D. melanogaster*. According to the HD formula as well as the intuitive definition of diversity, the “evenly distributed” haplotypes will produce a higher HD than the case where all individuals are skewed to a particular “dominant” haplotype. Therefore, we calculated the haplotype frequency for each pair of nonsynonymous editing sites. In pooled samples of *A. thaliana*, the CT and TC haplotypes are strongly suppressed while TT haplotype is dominant (Fig. 5B). This indicates that the C-to-U(T) events on two nearby editing sites are always linked: “all edited or none edited”, resembling the “all or none” property of DNA mutations. In contrast, in *D. melanogaster*, (1) the frequencies of AG, GA, and GG haplotypes are comparable, and (2) the frequency of AA is only slightly higher than the frequencies of other three haplotypes (Fig. 5B). We believe that the strong linkage between C-to-U editing sites causes the skewed distribution of haplotype frequency and consequently leads to low haplotype diversity.

Here we give two representative examples to show the sharp difference between the haplotype diversities of plant C-to-U and animal A-to-I RNA editing. In *D. melanogaster*, two nearby nonsynonymous editing sites (chr2R:9,234,514 and chr2R:9,234,519) of synaptic gene *hig* (*hikaru genki*) produce a combination of four protein isoforms, amplifying the neural proteomic diversity (HD = 0.63) in synapses (Fig. 5C). In *A. thaliana*, two adjacent nonsynonymous editing sites in mitochondrial gene ATMG00990 (*NAD3*) only have the all edited TT haplotype and the none edited CC haplotype in all tissues (Fig. 5D). The “single editing” CT and TC haplotypes were not detected. Given that the two editing sites are completely linked, the HD is extremely low across all tissues (Fig. 5D).

Intriguingly, strong linkage is also a representative feature for DNA mutations: for instance, two DNA mutations will always be linked in the RNA molecules; compared to the random RNA editing events on two nearby editing sites (which are weakly linked), DNA mutations have 100% linkage. Thus, the fact that C-to-U editing in *A. thaliana* has stronger linkage than A-to-I editing in *D. melanogaster* further proves that C-to-U editing in plants behaves like DNA mutations. DNA mutations are hard-wired and are not able to increase proteomic diversity in a spatial-temporal manner. Instead, DNA mutations are only able to fix genomic sequences if the current nucleotide is unfavorable (or less optimal than the ancient sequence). These observations and implications all support the restorative hypothesis but not diversifying hypothesis of C-to-U RNA editing in plants.

### Different routes to diversity: synergistic effect between editing and splicing?

**Fig. 6:**
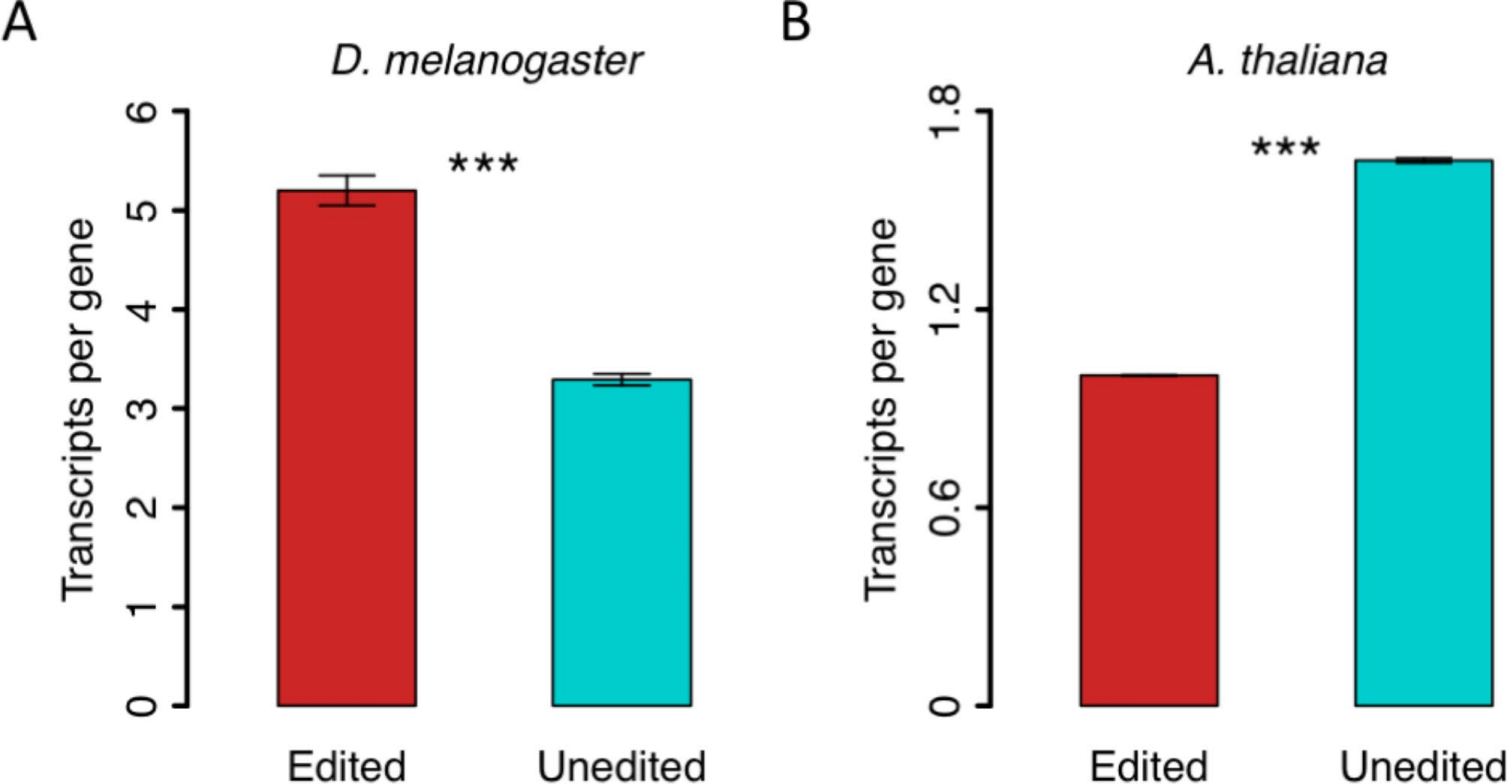
Numbers of transcripts per gene according to the annotation of the reference genome. (**A**) *D. melanogaster*. (**B**) *A. thaliana*. Error bars represent standard error of mean. *P* values were calculated using Wilcoxon rank sum tests. ***, *P* < 0.001

If RNA editing is used for diversifying the proteome, then it might interplay with other mechanisms that increase the proteomic complexity. Alternative splicing is such a mechanism that produces different protein isoforms. In *Drosophila*, it was reported that alternative splicing is prevalent in the neuronal genes to create various versions of transcripts [42]. Since A-to-I RNA editing is also abundant in *Drosophila* nerve systems, it is not surprising to observe that edited genes on average bear more isoforms than unedited genes (Fig. 6A). This pattern raises a possibility that RNA editing in *Drosophila* has synergistic effect with splicing to increase the transcriptomic and proteomic diversity.

In contrast, genes with C-to-U editing in *A. thaliana* are chloroplast and mitochondrial genes which only have one transcript per gene due to the unique nature of the non-nuclear genome. Therefore, it is expected to see that edited genes have less isoforms than unedited genes in *A. thaliana* (Fig. 6B). This again indicates that C-to-U RNA editing in plants is unlikely to be used for the diversifying purpose.

In *Drosophila*, neuronal genes might have the greatest demand for complex proteome so that A-to-I RNA editing and alternative splicing work together to achieve the neuron diversity. In plants, no such synergistic effect was seen between C-to-U RNA editing and splicing. However, the signal of adaptation is indeed observed in plant editome so there must be a plausible biological function of nonsynonymous C-to-U editing. Thus, the restorative hypothesis is the most possible explanation for the adaptiveness of recoding.

## Discussion

### Summary of main findings

Nonsynonymous RNA editing is either used for diversifying the proteome in a flexible manner (diversifying hypothesis) or for reversing unfavorable DNA mutations (restorative hypothesis). We observed signals of adaptation in the C-to-U RNA editomes of *A. thaliana* where nonsynonymous editing has higher occurrence and editing levels than synonymous editing. Editing levels in *A. thaliana* are sufficiently high and lack tissue-specificity, supporting the DNA mutation-like role of C-to-U RNA editing. We further calculated the haplotype diversity HD of nonsynonymous editing sites and compared the HD profiles between *A. thaliana* C-to-U editing and *Drosophila* A-to-I editing. Globally, C-to-U editing obviously has lower HD than A-to-I editing, presumably due to the strong linkage between C-to-U editing events. Therefore, C-to-U editing in *A. thaliana* is not designed for diversifying the proteome; instead, it acts like DNA mutations to reverse unfavorable genomic sequences. Our results for the first time verify the restorative hypothesis for plant C-to-U RNA editing by rejecting the alternative diversifying hypothesis. Moreover, we unprecedentedly improved the traditional nucleotide diversity θπ by defining the haplotype diversity HD of RNA molecules. Our ideas could be applied to a broad field of molecular biology, evolutionary genomics, and bioinformatics.

### Both diversifying and restorative hypotheses predict signal of adaptation

No matter nonsynonymous editing is designed for diversifying or restorative purpose, it has to be positively selected compared to synonymous editing. Therefore, signal of adaptation is expected under both hypotheses. Although the original restorative hypothesis states that the edited allele is no better than the ancestral DNA allele, it is intuitive to think that the edited allele is better than the unedited allele in extant species [11]. Then, the editing mechanism itself is certainly adaptive. To distinguish between diversifying hypothesis and restorative hypothesis, direct measurement and comparison of the proteomic diversity introduced by nonsynonymous editing will be helpful. Moreover, since the restorative hypothesis requires the editing sites to act like DNA mutations, the “all or none” property of DNA mutation could also be used as a criterion to judge whether the editing events really mimic genomic mutations.

Notably, both diversifying and restorative hypotheses only focus on nonsynonymous editing. There are many non-coding editing sites in repeat regions that function as anti-virus defense in mammals [8]. These editing sites are still functional although they are lowly edited and poorly conserved across species. Further quantitative approaches are needed to measure the adaptiveness of non-coding editing events.

### Limitation of the HD algorithm

HD is strongly affected by the number of mutation sites. In this study, we only considered pairs of nonsynonymous C-to-U editing sites (then the number of all possible combinations m = 4). If we extend the HD formula to X editing sites, then m = 2^X^. Obviously, m will drastically increase with X. When the sequencing coverage is not deep enough, the observed number of haplotypes (n) might be small and n/m will be even smaller. This will significantly reduce the HD value. HD values under different X (number of mutation sites) are not comparable. Therefore, this current HD formula needs to be refined to adapt to a broader range of cases. Nevertheless, the HD values are comparable when given the same number of mutation sites. The specific cases shown in our study prove that our HD parameter accurately reflects the proteomic diversity.

## Conclusions

C-to-U RNA editing in *Arabidopsis* is adaptive but it is not designed for diversifying the proteome like A-to-I editing in *Drosophila*. Instead, C-to-U recoding sites resemble DNA mutations. Our observation supports the restorative hypothesis of plant C-to-U editing which claims that editing is used for fixing unfavorable genomic sequences.

### Electronic supplementary material

Below is the link to the electronic supplementary material.

Supplementary Material 1

## Data Availability

We downloaded the RNA-Seq data of different tissues of *Arabidopsis thaliana* from NCBI with the following accession IDs: anther (SRR7734398 & SRR7734399), mature ovule (SRR8732884), petal of mature flower (SRR3581688 & SRR3581854), sepal of mature flower (SRR3581689 & SRR3581855), leaf (SRR2060715 & SRR2060717) and root (SRR3498212, SRR3498213, SRR3498214). The reference genome of *A. thaliana* was downloaded from Ensembl (genome version TAIR10). The A-to-I RNA editing sites in brains of *Drosophila melanogaster* were retrieved from our previous work [10]. The mRNA-Seq data of developmental stages and tissues of *D. melanogaster* were downloaded from NCBI with the following accession numbers: embryo 0-2 h (SRR5075630), embryo 2-6 h (SRR5075635), embryo 6-12 h (SRR5075633), embryo 12-24 h (SRR5075631), larva (SRR5075634), pupa (SRR5075632), female adult body (SRR3031117 & SRR3031118), male adult body (SRR3031119 & SRR3031120), female adult head (SRR7262145) and male adult head (SRR7262144). The honeybee (*Apis mellifera*) data and the matched editing sites were retrieved from our previous work [30].
